# Rifampin‐induced acute kidney injury and hemolysis: A case report and literature review of a rare condition

**DOI:** 10.1002/ccr3.6780

**Published:** 2022-12-21

**Authors:** Fateen Ata, Hiba M. B. Magboul, Haneen A. A. Toba, Hadeel Alfar, Adel Al Bozom, Khaled Murshed, Muhammad Zahid

**Affiliations:** ^1^ Department of Endocrinology Hamad Medical Corporation Doha Qatar; ^2^ Department of Internal Medicine Hamad Medical Corporation Doha Qatar; ^3^ Department of Anatomic Pathology Hamad Medical Corporation Doha Qatar; ^4^ Weill Cornell Medicine Doha Qatar; ^5^ College of Medicine Qatar University Doha Qatar

**Keywords:** acute interstitial nephritis, acute kidney injury, acute tubular necrosis, rifampicin, rifampin

## Abstract

Rifampicin is a bactericidal drug used in various infectious diseases, including tuberculosis (TB). Nephrotoxicity is a rare side effect of intermittent Rifampin use and even less commonly continued use. We report a case of Rifampin‐induced acute tubular necrosis and hemolysis in a patient with latent TB with a relevant literature review.

## Abstract 2

Acute kidney injury with hemolysis due to Rifampin (intermittent or continuous use) is rare and mainly presents as acute tubular necrosis or acute interstitial nephritis. The mechanism of injury is probably autoimmune‐mediated, with a good recovery.

## 1 INTRODUCTION

Rifampicin has been a widely used antibiotic drug to treat bacterial infections, including tuberculous and non‐tuberculous infections, since the 1970s.^1^ In addition to common adverse drug reactions such as hepatotoxicity, it is also associated with rare adverse effects, including renal injury. Although rare, several reports have been published describing the post‐rifampicin acute renal failure dating back to 1976.^2^ Most commonly manifesting as acute tubular necrosis (ATN) following re‐administration of rifampicin; it is postulated that an immune response likely plays a role in the development of the renal failure.^3^ Although reported multiple times, there are insufficient data to determine which patients are prone to renal failure post‐rifampicin use and how to manage this adverse reaction. It is noted that patients taking rifampicin also may develop hemolysis, with or without renal injury.^4^ This article will review a rare case report of kidney failure associated with hemolytic anemia and hepatitis following first‐time administration of rifampicin and a literature review of similar articles.

## 2 CASE REPORT

A 42‐year‐old Moroccan lady with no chronic comorbidities presented to the hospital with a 4‐day history of repeated vomiting, chills, bilateral flank pain, and epigastric discomfort. Her vomiting was non‐projectile, non‐bilious, and without blood. She also reported a yellowish skin discoloration and red discoloration in her urine. Upon examination, her vital signs revealed a temperature of 37.8°C, a heart rate of 77 beats per minute, a blood pressure of 119/81 mmHg, and oxygen saturation of 97% on room air. Scleral and skin icterus were noticed with mild generalized abdominal tenderness. The tenderness was present on deep palpation. No organomegaly was noted on the abdominal exam. Her respiratory, neurological, and cardiovascular examinations were unremarkable.

The patient was recently started on Rifampicin and Isoniazid (INH) for treatment of latent tuberculosis (LTB), evidenced by a positive PPD (21 mm) test with abnormal Chest Xray (which were performed as part of screening tests during pre‐employment examination). Her sputum smear for acid‐fast bacilli (AFB) and polymerase chain reaction (PCR) for Mycobacterium tuberculosis (MTB) at that time was negative, hence ruling out an active infection. She was a non‐smoker and did not consume alcohol. She was not taking any other medications and had no history of medication or food allergies.

Initial laboratory investigations revealed leukocytosis, anemia, transaminitis with direct hyperbilirubinemia, and renal function derangement (Table 1). The urine dipstick was positive for blood, protein, leukocytes, and nitrates. Urine microscopy showed elevated red and white blood cells with urine cast present but absent urine eosinophils (Table 2).

**TABLE 1:** Initial laboratory investigations of the patient including blood counts and metabolic panel

Labs	Upon admission	During overload period	After discharge
WBC	14.7	12.8	7.5
Hb	9.9 g/dl	5.2 g/dl	12.7 g/dl
Platelets	121	135	343
Creatinine	110 μmol/L	607 μmol/L	60 μmol/L
ALT	40 U/L	27 U/L	29 U/L
Total bilirubin	152 μmoL/L	117 μmol/L	6 μmol/L

**TABLE 2:** Urine analysis results upon admission

Urine workup	Results
Specific gravity	1.005
Proteins	3+
Bilirubin	3+
RBCs	14
WBCs	77
Casts	Granular casts

A provisional diagnosis of possible intra‐abdominal infection was made; however, blood cultures and hepatitis workup were negative. Upon further evaluation, evidence of hemolysis with features of acute kidney injury (AKI) was noted. Lactate dehydrogenase (1359 U/L, normal range: 135–214 U/L) and total bilirubin (152 μmol/L, normal range: 0–21 μmol/L), were elevated, with a low haptoglobin (<10 mg/dl, normal range: 30–200 mg/dl). Additionally, a direct antiglobulin test was performed, which was positive. The ultrasound of the urinary tract showed normal kidneys with no evidence of hydronephrosis, thus ruling out obstructive uropathy. Her AKI was not improving with the administration of intravenous fluids, and granular casts were present on urine microscopy, pointing toward tubular injury. Further investigations for AKI revealed normal C3 and C4 levels with negative antinuclear and antineutrophil cytoplasmic antibodies. MTB Workup was repeated, which also came back negative.

After ruling out pre‐renal causes (no hypovolemia and absence of septic shock), obstructive causes (no evidence of obstruction on imaging), and with supporting clinical and laboratory picture, a diagnosis of drug‐induced ATN and hemolytic anemia was made.

The patient was given an intravenous fluid challenge without an improvement in kidney function. Rifampicin was considered the potential cause of the ATN. As initial management, Rifampicin and INH were suspended on the third day of her admission, and the patient was started on IV methylprednisolone (250 mg for 3 days), which was then changed to oral prednisolone 60 mg (1 mg/kg) with a tapering schedule over 4 weeks.

**FIGURE 1:**
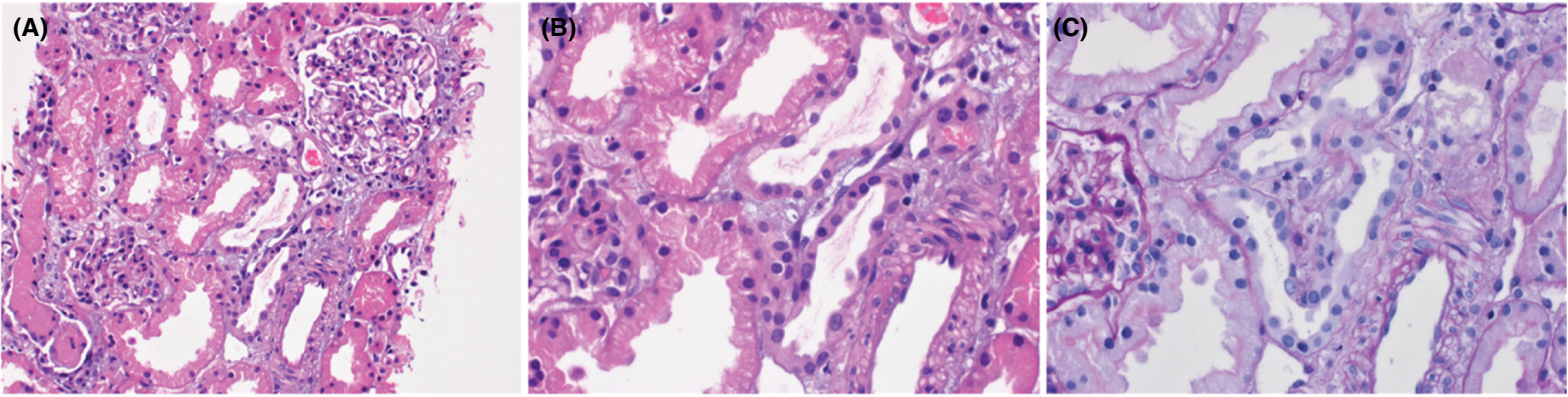
(A) Photomicrograph shows renal parenchyma in which some of the tubules have flattened epithelium, indicating acute tubular injury (Hematoxylin & Eosin ×200). (B) High power view shows that two tubules have flattened epithelium. They are lined by cells that have large nuclei with prominent nucleoli indicating regenerative changes (Hematoxylin & Eosin ×400). (C) High power view shows three tubules in the center that have flattened epithelium with loss of brush border. (Periodic Acid‐Schiff stain ×400.)

During her hospital course patient developed anuria and volume overload with a sharp rise in her serum creatinine levels so hemodialysis was commenced. A Kidney biopsy showed evidence of ATN with evidence of regeneration and mild AIN (Figure 1).

The patient's kidney functions started to improve gradually, and the patient started passing a good amount of urine after steroids and two sessions of hemodialysis. The patient was discharged in an asymptomatic condition without any need for long‐term renal replacement therapy. A follow‐up visit 3 weeks after discharge showed normal kidney function. The patient was followed up in the TB clinic. Anti‐tubercular treatment was not resumed as her repeated tests, including Acid‐fast bacilli smears, MTB PCR and QuantiFERON, were negative.

## 3 DISCUSSION

Tuberculosis, a curable and preventable disease, affected 10 million people, with 1.5 million deaths worldwide in 2020.^5^ Rifampicin is a widely used drug, most commonly in treating pulmonary tuberculosis.^1^ AKI is a rare complication in anti‐tuberculosis therapy (ATT) patients. Although ethambutol and INH have been associated with AKI, most studies reveal rifampicin as the commonest associated drug among the patients who developed AKI while on ATT.^6^, ^7^, ^8^, ^9^ Rifampicin‐associated AKI is reported in <0.1% of patients with TB. It has also been reported in the setting of leprosy, staphylococcal endocarditis, and infection with non‐tuberculous mycobacteria.^7^, ^10^, ^11^, ^12^, ^13^ The Literature review reveals that most patients with rifampicin‐induced AKI might also develop concomitant hemolytic anemia.^4^

The definition of rifampin‐associated AKI varies in different studies, and the exact incidence rate remains unknown. One review article from Romania reports that 0.05% of patients receiving rifampicin (mean age, 45 years) developed AKI, which was defined as elevated serum creatinine >44.2 μmol/L or > 20% of baseline in 2 weeks.^14^ We conducted a brief review of previously reported cases in the literature entailing rifampicin‐induced AKI or hemolytic anemia (alone or in combination). With a systematic literature review of previously published reports in PubMed, Scopus, and Google Scholar (from any date to 1st November 2021), we could identify 25 Rifampin‐induced AKI or hemolytic anemia cases (Appendix S1). The mean age of patients was 47.7 years, with the majority being men (72%) (Table 3). The gender difference can mainly be justified by the fact that TB itself is much more prevalent in men than women.^15^ It was previously thought that the reason behind this gender difference could be limited access to healthcare for women, resulting in an undiagnosed disease burden in females. However, studies have shown that the gender difference in TB prevalence is real when such covariates are balanced among both groups.^16^ This difference might have translated into our analysis because 69.2% of patients who took rifampicin and developed AKI had TB (pulmonary and abdominal TB) as their diagnosis. As expected, various other diseases indicating rifampicin use were identified, including leprosy, brucellosis, methicillin‐resistant staphylococcus aureus (MRSA) bloodstream infection, and mycobacterium avium complex (MAC). Out of the 25 cases, AKI was observed in 23 patients, whereas hemolytic anemia was reported in 16. Various types of AKI were reported as tabulated (Table 3). Noncompliance to rifampicin, also termed intermittent use, was found in 36% of cases. This result was deemed clinically significant because previously, it was thought that rifampicin‐induced AKI is seen mainly in these patients. However, our review reveals that a majority of cases (69.2%) had continuous rifampicin use, which led to AKI. Our literature review shows that AKI secondary to rifampicin use has a good recovery rate (88%). Although this data is clinically significant and relevant in an era where we still fight TB actively in a large part of the globe, it needs a detailed and extensive analysis in a larger patient population.

**TABLE 3:** Clinical characteristics of patients reported in the literature who developed Acute kidney injury or hemolytic anemia (alone or in combination) with the use of rifampin.

Characteristics	Results (*N* = 25)
Mean age (in years)	47.7 ± 17.4
Gender	
Male	18/25 (72%)
Females	7/25 (28%)
Indications of Rifampin	
Pulmonary TB	15/25 (60%)
Leprosy	3/25 (12%)
Brucellosis	2/25 (8%)
MRSA infection	2/25 (8%)
Abdominal TB	2/25 (8%)
MAC	1/25 (4%)
Comorbidities	
Diabetes mellitus	2/25 (8%)
Lung disease	1/25 (4%)
Noncompliance to Rifampin	8/25 (32%)
Anti‐Rifa antibodies	8/25 (32%)
Acute kidney injury	23/25 (92%)
Tubular necrosis	8/25 (32%)
Glomerulonephritis	4/25 (16%)
Interstitial nephritis	3/25 (12%)
Type not specified	9/25 (36%)
Treatment of AKI	
Dialysis	9/25 (36%)
Discontinuation of Rifampin	7/25 (28%)
Steroids	2/25 (8%)
Outcomes of AKI	
Complete recovery	21/25 (84%)
Partial recovery	1/25 (4%)
Persistent proteinuria	2/25 (8%)
Hemolytic anemia	16/25 (64%)

Abbreviations: AKI, acute kidney injury; MAC, mycobacterium avium complex.

Data regarding the time of initiation of AKI after exposure to rifampicin is variable in the literature. The median interval between the start of anti‐TB treatment and the onset of AKI has been reported to be as long as 44 days in one study. Rifampicin is known most likely to cause AKI when it is used irregularly or reintroduced. Furthermore, it is also reported that the type of AKI might have some association with the therapy regimen; ATN seems to manifest more commonly after intermittent use, whereas acute interstitial nephritis (AIN) manifested with uninterrupted therapy.^17^ However, our case presented with ATN following continuous, uninterrupted rifampicin therapy for 10 days.

Symptoms of AKI secondary to rifampin are similar to AKI of any other cause. The most common presenting symptoms were anuria, gastrointestinal upset, and flu‐like symptoms.^7^ Our case presented with fulminant symptoms; predominant gastrointestinal symptoms, an oliguric course requiring dialysis, and anemia due to intravascular hemolysis.

The mechanism of rifampin‐associated AKI is not well established. Several studies suggest either a type II or type III hypersensitivity reaction induced by rifampin antigens in which anti‐rifampin antibodies form immune complexes deposited in renal vessels, the glomerular endothelium, and the interstitial area.^3^ During our literature review of previously published cases, Anti‐rifampin antibodies were found to be positive in 32% (Table 3). In a retrospective study by Covic et al., more than half of the patients developed hemolytic anemia, with 17% having hepatic dysfunction^7^; all these points favor an immune‐mediated origin of kidney impairment in patients taking rifampin. However, this is yet to be validated in more extensive prospective studies. The deposition of immune complexes in the vessels causes vascular constriction and tubular ischemia, leading to ATN, whereas the deposition of immune complexes in the interstitial area leads to AIN. Renal biopsies performed in several studies with a total of 106 patients reveal that the most common pathologies are AIN (54%) and ATN (38%).^7^, ^10^, ^11^, ^12^, ^13^ A kidney biopsy was done in our case and showed evidence of ATN with evidence of regeneration and mild AIN.

Various mechanisms of drug‐induced immune‐mediated hemolytic anemia (DIIHA) have been proposed. DIIHA‐related antibodies are either drug‐dependent (react to RBCs in the presence of the drug) or drug‐independent (react to RBCs without the presence of the offending drug).^18^ Exposure to rifampicin leads to the formation of IgM and IgG antibodies, which interact with I‐antigen expressed on the surface of RBCs leading to complement‐mediated RBC hemolysis. The I‐antigen is also expressed in renal tubular cells mediating immune injury.^6^ Rifampicin‐related IgG or IgM antibodies causing RBC lysis, platelet lysis and renal tubular cell damage are mainly observed with the intermittent or interrupted use of rifampicin.^19^

In previous studies, more than 80% of patients recovered from AKI within 120 days.^7^, ^10^, ^11^, ^12^, ^13^ 36% of patients reviewed in the articles required dialysis (Table 3). Additionally, a good proportion of patients responded well to steroids, the median starting steroid dose was 50 mg (range: 40–50 mg), and the median duration of steroid administration was 52 days.^20^ These studies show a complete recovery of kidney function after treatment in most patients with an excellent long‐term prognosis.^7^, ^8^ It is recommended not to resume rifampicin for any patient who developed AIN.^20^ Our patient required steroids (for 3 months) and hemodialysis sessions, and her kidney functions normalized 3 weeks after discontinuation of the culprit drug, in keeping with the previous cases reported in the literature.

## 4 CONCLUSIONS

Acute kidney injury is a rare complication associated with rifampicin use, challenging its recognition. This article reported a rare case of rifampicin associated with ATN following the first‐time uninterrupted introduction of rifampicin. Associated presence of hepatitis, hemolytic anemia, and some reports of the presence of Anti‐rifampicin antibodies point toward an immune‐mediated mechanism of AKI. Therefore, patients developing AKI while on rifampicin should have this important differential diagnosis in mind, as discontinuation of the drug results in a favorable prognosis in most cases.

## AUTHOR CONTRIBUTIONS

**Fateen Ata:** Conceptualization; data curation; formal analysis; investigation; methodology; project administration; validation; writing – original draft; writing – review and editing. **Hiba M. B. Magboul:** Data curation; investigation; writing – original draft. **Haneen A. A. Toba:** Data curation; investigation; writing – original draft. **Hadeel Alfar:** Data curation; investigation; writing – original draft. **Adel Al Bozom:** Data curation; investigation; writing – original draft. **Khaled Murshed:** Data curation; investigation; writing – original draft. **Muhammad Zahid:** Conceptualization; data curation; supervision; validation; writing – review and editing.

## CONFLICT OF INTEREST

The authors declare that they have no competing interests.

## ETHICAL APPROVAL

Ethical approval for this study was obtained from Medical Research Center (MRC) Qatar (MRC‐04‐22‐287).

## CONSENT

Written consent was taken from the patient and her mother for publication of data and accompanying images.

## Supporting information


Appendix S1.
