# Supplementary material for: Rifampin‐induced acute kidney injury and hemolysis: A case report and literature review of a rare condition
Source: Clin Case Rep. 2022 Dec 21;10(12):e6780. doi: 10.1002/ccr3.6780 (PMC9771787; doi:10.1002/ccr3.6780)
Supplement: Supplementary file 1 — Appendix S1. [file CCR3-10-e6780-s001.docx]

Added cases in the manuscript ^1-19^

1. Levine, M., Collin, K. & Kassen, B. O. Acute hemolysis and renal failure following discontinuous use of rifampin. *Dicp* **25**, 743-744, doi:10.1177/106002809102500709 (1991).
2. Tahan, S. R., Diamond, J. R., Blank, J. M. & Horan, R. F. Acute hemolysis and renal failure with rifampicin-dependent antibodies after discontinuous administration. *Transfusion* **25**, 124-127, doi:10.1046/j.1537-2995.1985.25285169202.x (1985).
3. Salih, S. B., Kharal, M., Qahtani, M., Dahneem, L. & Nohair, S. Acute interstitial nephritis induced by intermittent use of rifampicin in patient with brucellosis. *Saudi J Kidney Dis Transpl* **19**, 450- 452 (2008).
4. Rosati, S. *et al.* Acute rifampicin-associated interstitial tubulopathy in a patient with pulmonary tuberculosis: a case report. *J Med Case Rep* **7**, 106, doi:10.1186/1752-1947-7-106 (2013).
5. Mauri, J. M. *et al.* Antirifampicin antibodies in acute rifampicin-associated renal failure. *Nephron*

**31**, 177-179, doi:10.1159/000182639 (1982).

1. Gupta, A., Sakhuja, V., Gupta, K. L. & Chugh, K. S. Intravascular hemolysis and acute renal failure following intermittent rifampin therapy. *Int J Lepr Other Mycobact Dis* **60**, 185-188 (1992).
2. Neunert, C. E., Paranjape, G. S., Cameron, S. & Rogers, Z. R. Intravascular hemolysis following low dose daily rifampin. *Pediatr Blood Cancer* **51**, 821-823, doi:10.1002/pbc.21709 (2008).
3. Cheng, J. T. & Kahn, T. Potassium wasting and other renal tubular defects with rifampin nephrotoxicity. *Am J Nephrol* **4**, 379-382, doi:10.1159/000166858 (1984).
4. Murray, A. N., Cassidy, M. J. & Templecamp, C. Rapidly progressive glomerulonephritis associated with rifampicin therapy for pulmonary tuberculosis. *Nephron* **46**, 373-376, doi:10.1159/000184394 (1987).
5. Hirsch, D. J., Bia, F. J., Kashgarian, M. & Bia, M. J. Rapidly progressive glomerulonephritis during antituberculous therapy. *Am J Nephrol* **3**, 7-10, doi:10.1159/000166679 (1983).
6. Kora, R., Brodsky, S. V., Nadasdy, T., Agra, D. & Satoskar, A. A. Rifampicin in Nontuberculous Mycobacterial Infections: Acute Kidney Injury with Hemoglobin Casts. *Case Rep Nephrol* **2018**, 9321621, doi:10.1155/2018/9321621 (2018).
7. De Vriese, A. S., Robbrecht, D. L., Vanholder, R. C., Vogelaers, D. P. & Lameire, N. H. Rifampicin- associated acute renal failure: pathophysiologic, immunologic, and clinical features. *Am J Kidney Dis* **31**, 108-115, doi:10.1053/ajkd.1998.v31.pm9428460 (1998).
8. Chiba, S., Tsuchiya, K., Sakashita, H., Ito, E. & Inase, N. Rifampicin-induced acute kidney injury during the initial treatment for pulmonary tuberculosis: a case report and literature review. *Intern Med* **52**, 2457-2460, doi:10.2169/internalmedicine.52.0634 (2013).
9. Abu-Romeh, S. *et al.* Rifampicin-Induced Acute Renal Failure: A Case Report. *Saudi Journal of Kidney Diseases and Transplantation* **7**, 401-403 (1996).
10. Grilo Novais, A., Silva, C., Coelho, A. R., Silva, R. & Carvalho, A. C. Rifampicin-Induced Nephrotoxicity in a Tuberculosis Patient: Treatment Dilemma? *Eur J Case Rep Intern Med* **8**, 002833, doi:10.12890/2021_002833 (2021).
11. Sveroni, D., Stefos, A., Rigopoulou, E. I. & Dalekos, G. N. Rifampicin: not always an innocent drug. *BMJ Case Rep* **11**, doi:10.1136/bcr-2018-227356 (2018).
12. Min, H. K. *et al.* Rifampin-associated tubulointersititial nephritis and Fanconi syndrome presenting as hypokalemic paralysis. *BMC Nephrol* **14**, 13, doi:10.1186/1471-2369-14-13 (2013).
13. Sanwal, C., Kaldas, A., Surani, S. & Bailey, M. Rifampin-Induced Acute Intravascular Hemolysis Leading to Heme Pigment-Related Kidney Injury. *Cureus* **12**, e9120, doi:10.7759/cureus.9120 (2020).
14. Namisato, M. & Ogawa, H. Serious side effects of rifampin on the course of WHO/MDT: a case report. *Int J Lepr Other Mycobact Dis* **68**, 277-282 (2000).
